# CBCT artefact-burden of zirconia-based as compared to titanium implants for different beam energies: an analytical approach

**DOI:** 10.1038/s41598-022-19379-y

**Published:** 2022-09-10

**Authors:** Ralf Schulze

**Affiliations:** Division of Oral Diagnostic Sciences, Dental School, University of Bern, Bern, 3010 Switzerland

**Keywords:** Biophysics, Medical research, Biomedical engineering

## Abstract

Beam hardening artefacts induced by highly-dense material (e.g. metal) is a common quality issue in maxillofacial Cone Beam Computed Tomography (CBCT-) images. This experimental and analytical study investigated attenuation patterns of two typical dental implant materials: zirconia-ceramic and pure titanium. By application of different x-ray beam energies (60, 70, 80, 90 [kVp]) energy-dependent attenuation of these materials is assessed and the resulting artefact induction in the resulting CBCT-images evaluated. A zirconia (Y-TZP-) implant ($\varnothing$: 4.1 mm) and a pure titanium rod ($\varnothing$: 4.0 mm) were exposed in a commercial CBCT (3D Accuitomo 170). The raw two-dimensional (2D) projection radiographs the CBCT utilizes for three-dimensional reconstruction applied for acquisition of attenuation profiles through the circular central slice of the implant-phantom images. Distances the x-rays traverse through the implant-phantoms at this location were computed. Using this information and the linear attenuation coefficient, transmission and attenuation was computed for each material and beam energy. These data were related to beam hardening artefacts that were assessed in the axial reconstructions of the implants’ CBCT images. Transmission of titanium for all peak kilovoltages (kVp) was higher and approximately 200% that of Y-TZP at 60 kVp versus 530% at 90 kVp. At 4 mm diameter transmission for Y-TZP was only approximately 5 % for all four beam-energies. In agreement with this finding, beam hardening artefacts for Y-TZP could not be reduced using higher energies, whereas for titanium they decreased with increasing energy. For the energy spectrum used in this study (60–90 kVp), beam hardening caused by titanium can be reduced using higher energies while this is not the case for zirconia-ceramic (Y-TZP).

## Introduction

Cone Beam Computed Tomography (CBCT) plays an important role in preoperative planning of dental implants^1^. As implants have become a common restoration in many countries, often they will also be depicted in CBCT-scans acquired from their carriers. The vast majority of dental implants is made of highly pure Titanium with a chemically very stable oxide-surface^2^. Their artefact influence in CBCT scans has been experimentally described^3–5^ and also analytically explained^6^. In recent years, dental implants made of zirconia are increasingly marketed and inserted^7^. These are made of crystalline zirconium dioxide generally stabilized with 3 mol% Yttrium (Y-TZP)^8^. The dimensions and designs are roughly the same as in titanium implants. The fundamental part of beam hardening artefacts occur due to the filtering effect of the highly-dense, i.e. severely attenuating implant body which changes the energy-spectrum of the x-ray beam. After penetration of such highly-dense (strongly attenuating) bodies the beam contains relatively more higher-energetic (shorter wavelength) x-rays than the spectrum emitted by the source. This process is termed beam hardening. Unfortunately, the reconstruction assumes identical energies in the spectra and this error propagates into the three-dimensional (3D) reconstruction process^9^. Briefly explained, the relatively too high energy arriving at the detector “behind” the attenuating object (here: the implant) is being backprojected into the reconstructed volume, resulting in dark (hypo-dense) lines in beam direction. Attenuation is mainly caused by two main mechanisms: the Compton scattering and the photoelectric effect^10^. While the former is rather stable over the energy spectrum typically applied for CBCT, the photoelectric effect is strongly energy-dependent^10^. Here, the energy of the beam is capable to remove an electron from the inner k-orbit thereby producing a ion with a positive charge. At energies just above the required energy to remove the electron from the k-orbit of the respective material there is an abrupt increase of the attenuation of the material. This so-called “k-edge” is material-specific and increasing with increasing atomic number. The k-edge attenuation energy of the two materials investigated here is 4966.4 eV for Titanium versus 17,997.6 eV for Zirconium as main compound of zirconia-ceramic. Empirical studies investigating artefacts in CBCT-images caused by zirconia have been published^11–13^. However, to better understand the background and to potentially minimize their effects, these artefacts should also be studied from an analytical perspective using physical data and knowledge on the 3D reconstruction process.

Despite the rather large body of literature existing for experimental data on titanium and zirconia-ceramic (Y-TZP) CBCT-artefacts, to the best of our knowledge no analytical data on the underlying attenuation differences and their effect on the artefacts for different beam energies have been published so far. This work intended to discuss the background and investigate the artefact burden arising from zirconia-(Y-TZP-) and titanium-implants when imaged in a CBCT-machine using different beam energies. For this purpose, from the projection radiographs used for CBCT-reconstruction, the actual attenuation was computed for both materials and four different beam energies. The results of the attenuation experiments subsequently were compared to the beam hardening artefacts measured in the reconstructed implant images.

## Methods

### Theoretical background

**Figure 1:**
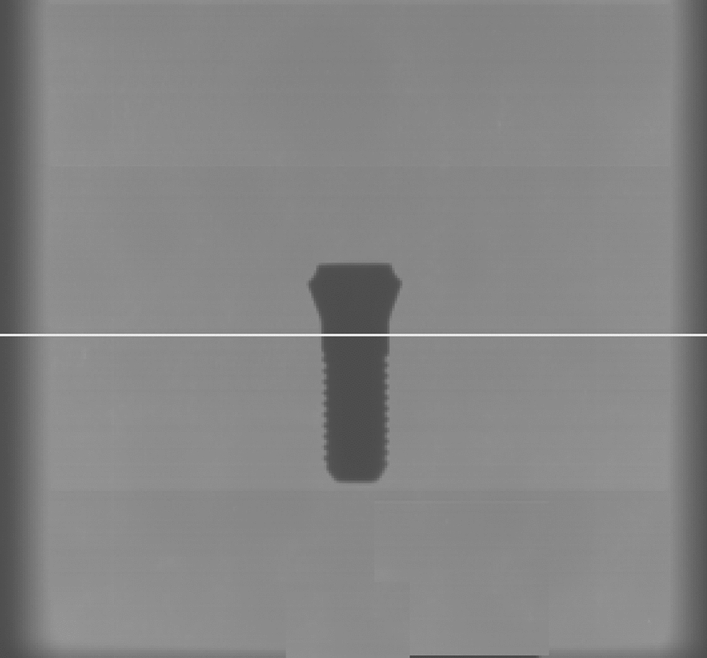
Exemplary 2D projection radiograph from the 3D Accuitomo with the Y-TZP-implant positioned in the center of the FOV. The white line represents the line along which the gray value profile was measured and evaluated, so that in eventually only the values within the implant image were used for computing transmission.

Beam-hardening artefacts are caused by filtering the longer wavelengths out of the emitted x-ray spectrum so that the beam “behind” (in the path of the x-ray beam) the filtering object (e.g. a dental implant) significantly differs from the emitted beam in composition. Since it only contains the shorter (higher energetic) wavelengths, the beam is “hardened”. Unfortunately, the reconstruction process assumes equality between emitted and beam arriving at the detector. This discrepancy induces error in the reconstruction process visible as darker (combined with some brighter) lines and stripes that are always orientated in the direction of the x-ray-beam. The latter is the the opposite (inverse) direction of the reconstruction process which is based on (filtered) backprojection^9^. Titanium with an atomic number of 22 has been shown to considerably alter the x-ray spectrum for the typical energies (60–120 kVp) used in dentomaxillofacial CBCT-machines^6^. Zirconium as base-substance for zirconia-implants has an atomic number of 40, hence inevitably the beam-hardening effect will be more prominent when compared to titanium. Y-TZP-implants are composed of zirconium- and yttrium-oxide. Unfortunately no exact mass-attenuation coefficients are available from the National Institute of Standards and Technology Web site (physics.nist.gov). Hence, for an analytic evaluation on expectable artefacts caused by Y-TZP experimental attenuation measurements are required. We will derive the attenuation from zirconia and titanium from the 2D projection radiographs (PROJs) (Fig. 1) acquired in a CBCT machine during exposure. These digital radiographs are directly used for the 3D-reconstruction of the CBCT-volume. Unfortunately in digital radiography there is no direct relationship between gray-value and dose^14^. Thus a direct measurement of gray-values within the projection images used for 3D-reconstruction is not indicative for incident beam energy. This is due to in comparison to film an extremely wide dynamic range of digital receptors and due to the required processing of the images for display. In other words, the gray-value of a certain pixel finally displayed to the observer can only be examined in a relative fashion. Nevertheless, the basic idea behind this research is to still use gray-values measured within the projection images used for CBCT-reconstruction. By exposing low absorbing air in addition to Y-TZP or titanium, a “relative” attenuation coefficient can be computed which then can enter the evaluation regarding beam-hardening artefacts. As no information on the output intensity $I_{0}$ of the source is easily available, the approach assumes that the intensity hitting the detector where only air is in the path of the beam most closely approximates $I_{0}$. Absorbing air of roughly the same dimension is also in the beam-path for all other structures exposed, hence using gray-values within a zone of air versus those within the implant image seems to provide a reasonable approximation. This approach assumes linearity in the image processing by the manufacturer. Since the manufacturers use the intensity (gray) values measured in the detector from the multitude of PROJs directly for their reconstruction process, this is certainly a meaningful assumption.

#### Theoretical model

**Figure 2:**
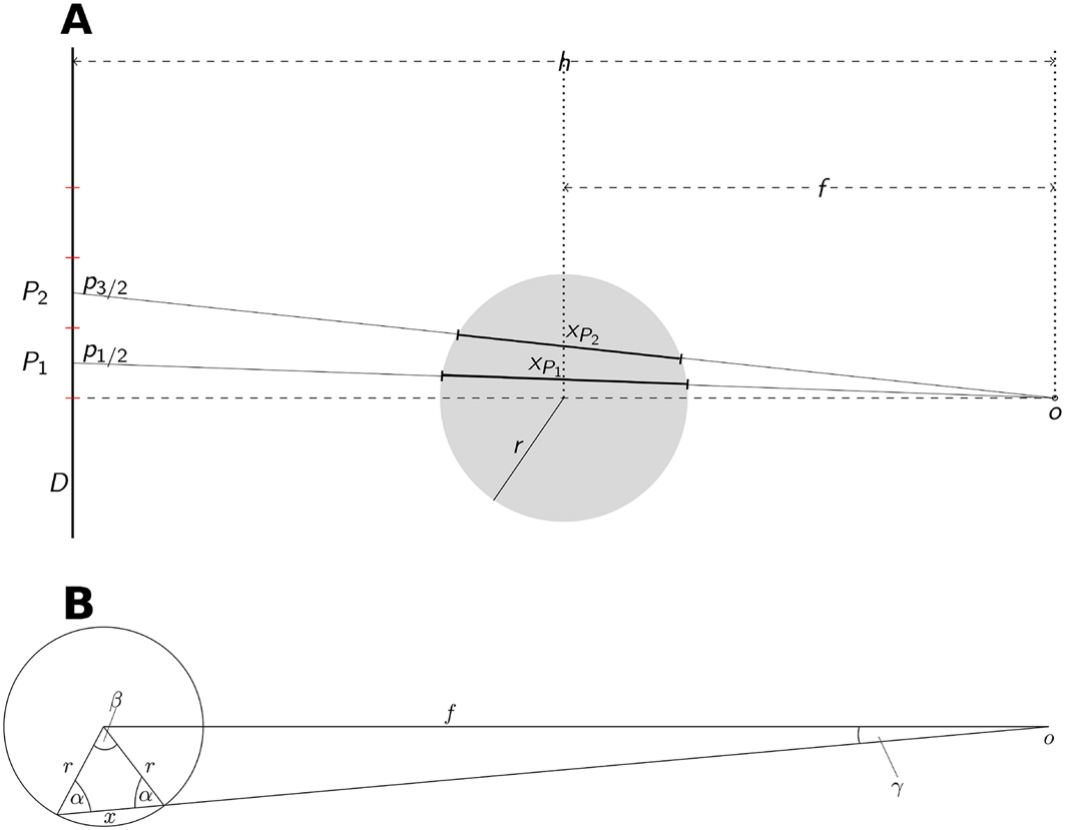
(**A**) Topview on the imaging geometry where the implant with radius *r* is exposed from the focal spot *o* on a detector *D*. The central x-ray is represented by the dashed line. Exemplarily the pixels $P_{1},P_{2}$ with dimension *p* and the respective distances $x_{P_{1}},x_{P_{2}}$ are indicated. Their centers are found at $p_{1/2},p_{3/2}$. (**B**) Distances and angles within the implant-cross-section used for calculation of the the x-ray-paths through the implant.

To investigate the proportion of transmitted (not attenuated) radiation, neglecting attenuation by air we assume that emitted radiation equals the incident radiation in areas where only air is depicted. The linear attenuation coefficient is obtained from:

1 $$\begin{aligned} I=I_{0}e^{-\mu \,x} \end{aligned}$$

which can be rewritten as

2 $$\begin{aligned} \mu =-\ln \frac{I}{I_{0}}\,x \end{aligned}$$

with *I* and $I_{0}$ representing incident and emitted intensity, respectively, and *x* the distance the x-rays traverse the absorber. $\mu$ in Eq. (2) denotes the “relative” mass attenuation coefficient under the assumption that the emitted intensity can be derived from the pixels in regions where only air was acting as absorber. The proportion of Intensity measured “behind” an absorber defines Transmission *T*, where $T=\frac{I}{I_{0}}$. After rearranging Eq. (2) and introducing the decimal logarithm we obtain the attenuation $A=\log \frac{I_{0}}{I}$. As absorber a dental implant is assumed with radius *r* positioned at a focal-object-distance *f* (known from specifications of the CBCT-manufacturer) from the point-shaped x-ray source *o* that is at a distance *h* from the detector *D* (Fig. 2A).

The model bases on the computation of the distance *x* the beam passes through the implant (Fig. 2) in the part of the implant image, which represents the circular cross-section of the implant, i.e. that part which is traversed by the central x-ray. This distance *x* is given by

3 $$\begin{aligned} x=2r\cos \left( \arcsin \left( \frac{\sin \gamma f}{r}\right) \right) \end{aligned}$$

for all angles $\gamma \in \left( 0,\gamma _{max}\right)$ (Fig. 2B) with:

4 $$\begin{aligned} \gamma _{max}=\arctan \frac{r}{d} \end{aligned}$$

where d denotes the distance from o to the tangential point where the x-ray touches the implant. We aim to obtain *x* for the pixels $P_{i}$ with $i\in (1,\ldots ,k)$ denoting the *k* Pixels with side-length *p* that are covered by the implant shadow (Fig. 2) when counted from the central x-ray ($\gamma =0$) into the periphery of the implants’ cross-section image. Consequently, the angle $\gamma _{P_{i}}$ between central x-ray and the *i*th pixel $P_{i}$ is obtained from:

5 $$\begin{aligned} \gamma _{P_{i}}=\arctan \frac{ip-0.5p}{h}, \end{aligned}$$

#### Image acquisition

**Figure 3:**
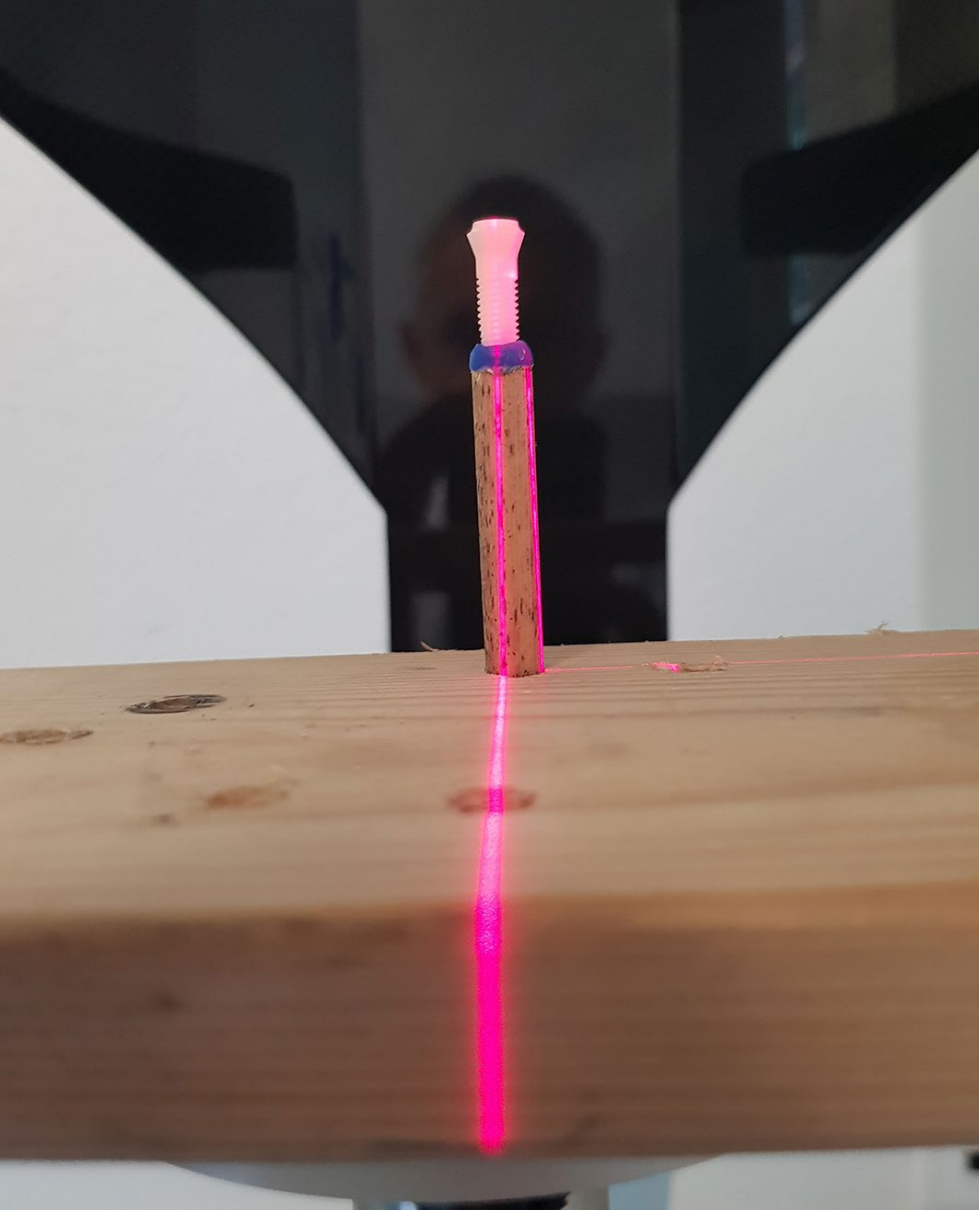
Set-up with the Y-TZP-implant centered and exposed in the CBCT-device. By means of the integrated laser-beam the central-ray was exactly centered in the field of view so that the required implant cross-section was centered in each PROJ.

CBCT scans were acquired from (a) an Y-TZP circonia implant (Patent, Zircon Medical Management AG Altendorf, Switzerland) of 4.1 mm diameter and a pure titanium rod (TICO, Titan Concept, Berlin, Germany) of diameter 4.0 mm. All exposure parameters (Table 1) were kept constant except for the four different beam energies (peak kilovoltages: 60, 70, 80, 90 [kVp], Table 1). CBCTs were acquired with the 3D Accuitomo 170- machine (J Morita Corp, Kyoto, Japan; internal filter: 3.1 mm aluminum) and standard exposure mode. For this purpose the implant was placed exactly vertically aligned fixed on a wooden rod in the center of the field of view (FOV), i.e. where the implant part used for the assessment was exactly centered within the field of view so that the central x-ray passes trough it. Under this assumption the line in Fig. 1 represents that plane where a circular cross-section of the implant is imaged. It should be noted, that only in this position the source-to-object-distance *f* as specified by the manufacturer (Table 1) is accurately applicable. This was ensured by means of the xyz-positioning-laser plus the scout function implemented in this CBCT-device. The latter enables accurate refinement of the initial laser-defined position by subsequently placing a mouse-driven aiming-rectangle on the monitor on the two perpendicular scout-radiographs. According to this refinement the machine corrects the position of the source-detector-unit relative to the object by means of motor-driven xyz-motion. The Y-TZP-implant coronally contains a cavern of approx. 3 mm depth for the abutment. Hence, the full-material part beneath that cavern yet above the thread had to be positioned in the center of the FOV to ensure that the full diameter of 4.1 mm Y-TZP was positioned in the location where the measurements subsequently were conducted in the projection images (Fig. 3). By accurately placing the measured part of the implants in the center of the FOV, the geometric assumptions described above (Fig. 2) are met as accurately as possible.

To assess repeatability, under identical conditions a second set of CBCTs was acquired several weeks after the first acquisition.

**Table 1:** Exposure settings for acquisition of the CBCT-scans.

Setting no.	kVp	mA	FOV-size [mm]	*f* [mm]	*h* [mm]	Pixel-size [mm]
1	60	5	40 $\times$ 40	540.0	842.0	0.254
2	70	5	40 $\times$ 40	540.0	842.0	0.254
3	80	5	40 $\times$ 40	540.0	842.0	0.254
2	90	5	40 $\times$ 40	540.0	842.0	0.254

From these scans only the 2D-projection images acquired under the exposition parameters specified above were used for further evaluation.

*mA* milliampere, *kVp* peak kilovoltage, *f* source-to-object-distance, *h* source-to-receptor-distance.

#### Image evaluation and computation of attenuation

The 3D Accuitomo allows for export of the 578 PROJs as multi layer tiff-file. The single tiff-files (projection radiographs) were extracted using ImageJ (https://imagej.nih.gov/ij/download.html) and saved in original 16 bit depth (example see Fig. 1). Discarding the first 50 projection radiographs which in the Accuitomo contain no image data, the remaining 528 PROJs were divided by 10 so that every 52th PROJ was extracted to ensure equally distributed images over the 360$^\circ$ scan-arch of the device. In each of the resulting 11 projection radiographs per kilovoltage a horizontal line through the entire radiograph was constructed through the implant-body (Fig. 1) in the vertical center of the radiograph at the same y-coordinate. The resulting gray-scale profile of that line was saved as numerical values. Due to unavoidable small positioning errors of the implant in the device the implant-profiles slightly differed in their x-coordinate position in the PROJs. To reduce such errors the 11 profiles obtained over the 360$^\circ$-range needed to be averaged. For this purpose, using R language and environment for statistical computing (R Foundation for Statistical Computing, Vienna, Austria) a numerical derivative of each profile was computed with its sharp maxima indicating the width of the implant-image in each line-profile. The profiles were symmetrically truncated on both sides of those maxima so that they exactly fitted to one another. In doing so, now only those values along the implant image were obtained which subsequently were averaged (11 profiles per voltage and material) to obtain a stable implant attenuation profile for further processing. In addition to the line-profile within the 11 PROJs per acquisition an area of 10 x 10 pixels in which only air was depicted was identified in the periphery roughly 1/4th from the image boundary. Since the Accuitomo integrates a non-removable carbonium-made headrest, for air-values no area was used containing the image of this headrest. These air-values were averaged over the 11 PROJs and defined as the maximum intensity $I_{0}$ incident on the detector for the respective kilovoltage. PROJs from both acquisition instances were evaluated in identical fashion.

**Computation of the distance** *x* Due to the known imaging geometry and pixel size on the detector the cord-length *x* defining the distance an “x-ray” detected in the center of a pixel travels through the implant. For this purpose only the angle $\gamma _{p_{i}}$ for the *i*th pixel $p_{i}$ can be computed and its’ value inserted into Eq. (4) to obtain the length of *x*. This is done for all pixels along the averaged line profile for each kilovoltage.

**Computation of beam-hardening artefacts in the reconstructed CBCT-data** The attenuation results were compared to the actual beam hardening occurring within the reconstructed implant phantom-images exported as DICOM-slices. Here, the reconstructed images of the first experiment were evaluated. To quantitatively compare beam hardening effects between the two materials and the energies, for all four energies line plots centered in the center of the cross-sections (Fig. 5) were generated extending over the edges of the implant image. This was done over a range of 170$^\circ$ so that 17plots were available per energy. These were averaged (per energy) and the numerical derivative for each mean profile computed. By using the maximum values of the first derivative the profiles were subsequently centered to one another and averaged. In doing so, one gray-value profile was computed for each beam energy (Fig. 6). In addition maximum relative differences between the central gray value within the implant images between 90 and 60 kVp were computed for both materials.

**Method error** Method error is described as difference in the computed transmission between the two acquisitions. These were evaluated by means of the paired T-test and a level of significance of 5%.

## Results

**Figure 4:**
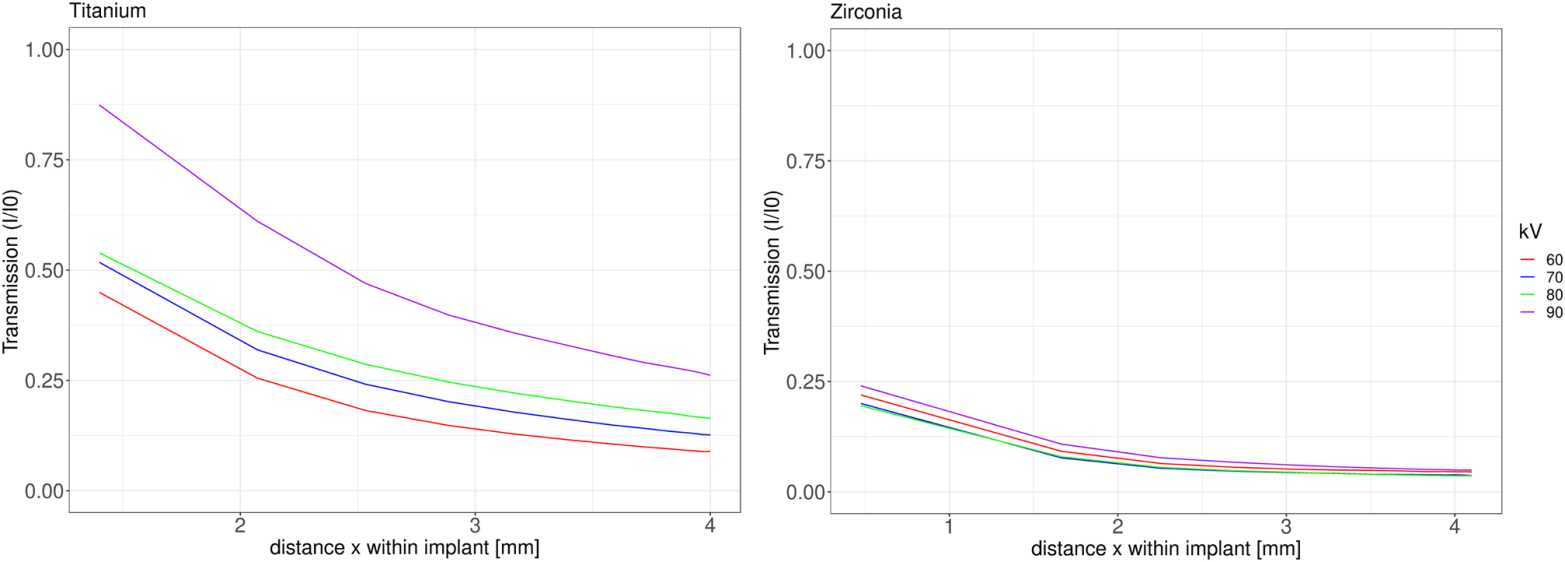
Transmission profiles for both materials and the four different beam energies averaged over both acquisitions. Note the transmission differences between the materials at equal distances within the material and the similarity of the transmission profiles for Y-TZP and all energies.

As to be expected, transmission *T* averaged over both acquisitions for the four kilovoltages was significantly higher for titanium than for Y-TZP (Fig. 4). For titanium, *T* at four millimeter distance *x* was 196% the transmission of Y-TZP at 60 kVp and 530% at 90 kVp (Fig. 4). *T* for Y-TZP at the full diameter of the implant (4.1 mm) for all four energies was relatively equal and only around 5% of $I_{0}$ (Fig. 4). For titanium the proportion of intensity impacting on the detector at x = 4 mm (implant diameter) is around 8% at 60 kVp and 26% at 90 kVp. For Y-TZP, the same range is very small with approximately 3–5% for all beam energies. From (Fig. 4) it is apparent that transmission for Y-TZP over all distances *x* within the implant is very similar for all beam energies. Regarding attenuation, for the 4 mm thick material a Y-TZP attenuated 68% more than titanium at 60 kVp, versus 225% more at 90 kVp (Table 2).

**Table 2:** Mean attenuation and transmission (± standard deviation) averaged over both experiments.

kVp	Parameter SD	Titanium	Y-TZP
60	Attenuation	0.8878379 ± 0.2141	1.2459 ± 0.1891
	Transmission	0.149 ± 0.1023	0.108 ± 0.0464
70	Attenuation	0.7509472 ± 0.1862	1.3222 ± 0.1990
	Transmission	0.1964 ± 0.1117	0.0877 ± 0.0431
80	Attenuation	0.6574 ± 0.1567	1.3270 ± 0.2015
	Transmission	± 0.1078	0.091 ± 0.0421
90	Attenuation	0.4492 ± 0.1616	1.1942 ± 0.1920
	Transmission	0.383 ± 0.1790	0.117 ± 0.0509

All values are computed over all intensities along the projection line within the implant image in Fig. 1.

### Method error

For neither of the energies a significant transmissiomn difference between first and second acquisition was observed (Table 3). Absolute differences between the transmission-values were small with absolute values ranging between − 0.00138 and 0.02813 for titanium versus 0.00589 and 0.02194 for Y-TZP (Table 3).

**Table 3:** Method error as absolute transmission differences between the two experiments.

kVp	Parameter	Titanium	Y-TZP
60	Absolute difference:mean$_{1,2}$	− 0.00546	− 0.00589
	p$_{\text {t-test}}$	0.9554	0.5353
70	Absolute difference:mean$_{1,2}$	− 0.00698	− 0.01925
	p$_{\text {t-test}}$	0.9924	0.9445
80	Absolute difference:mean$_{1,2}$	− 0.00138	− 0.02150
	p$_{\text {t-test}}$	0.957	0.9527
90	Absolute difference:mean$_{1,2}$	0.02813	− 0.02194
	p$_{\text {t-test}}$	0.6563	0.991

P-values (paired t-test) comparing Transmission in experiment 1 and 2 are also indicated. All values are computed over all intensities along the projection line within the implant image in Fig. 1.

### Impact on beam hardening

**Figure 5:**
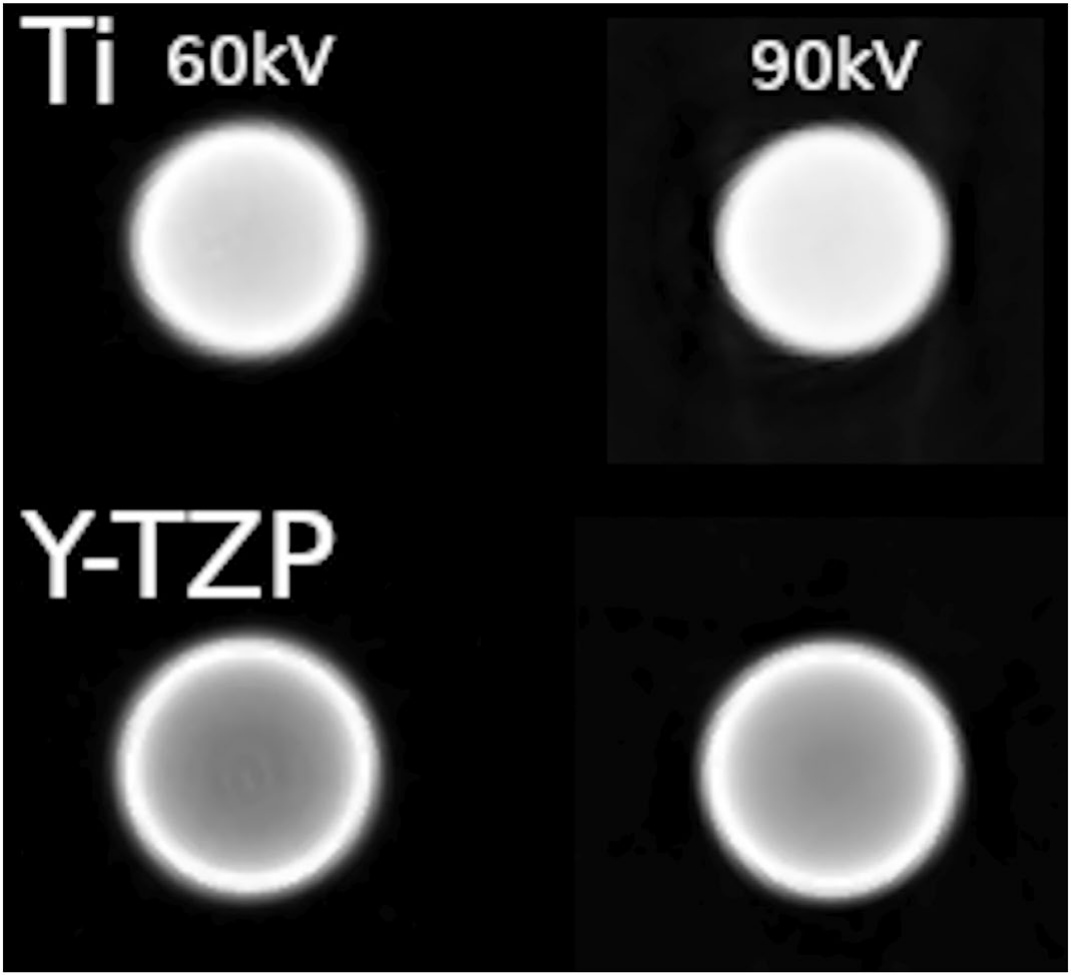
Slices through the DICOM CBCT-reconstruction of the implant samples for both materials and minimum (60 kVp) versus maximum (90 kVp) beam energy. Beam hardening is clearly visible in the center of the homogenouos implant as significantly darker gray values. This is particularly emphasized in the Y-TZP reconstructions (lower row).

**Figure 6:**
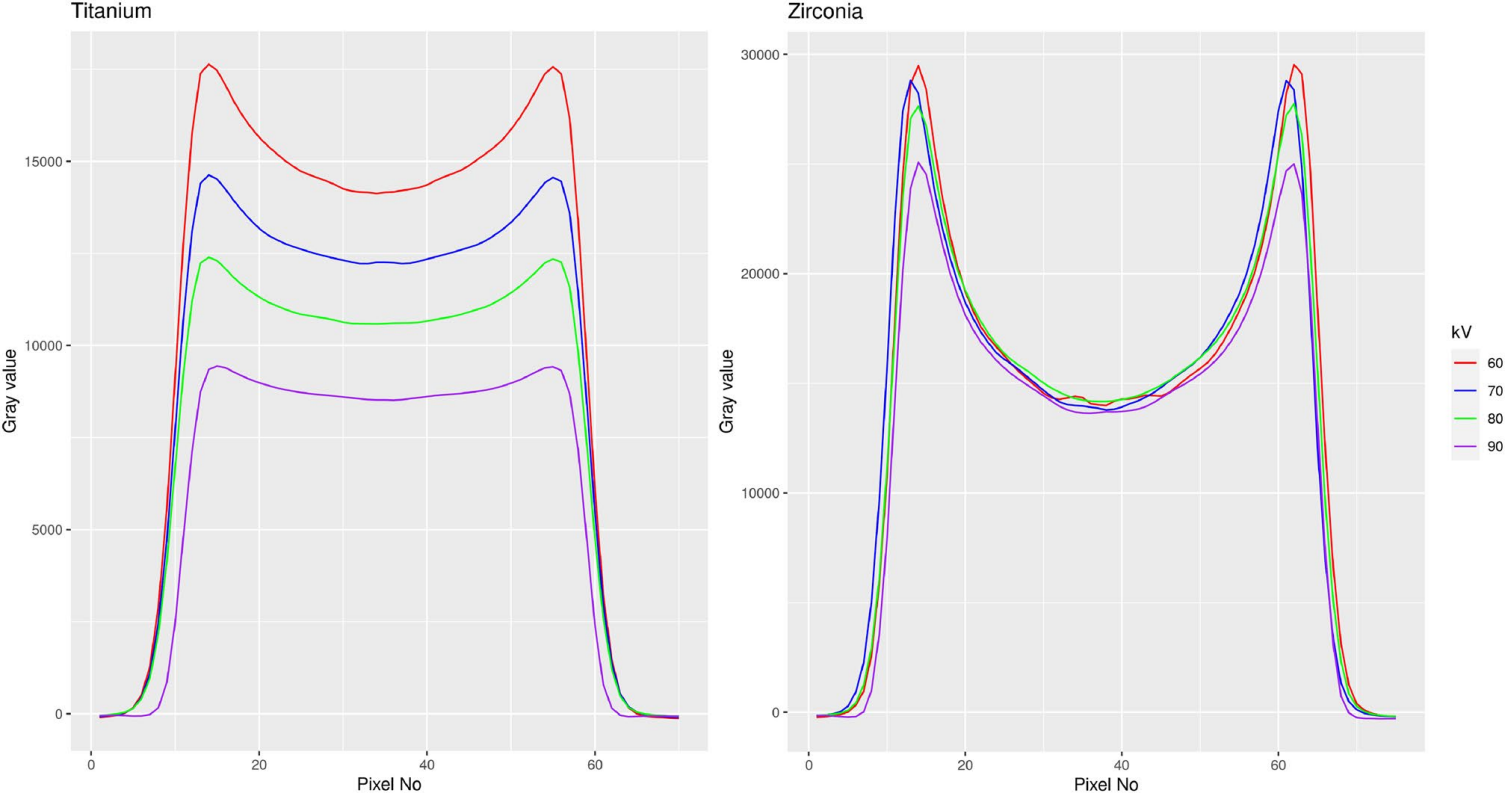
Numerical comparison of the actually occurring beam hardening in the reconstructed 3D CBCT-images of the implant samples. The plot shows averaged gray value-profiles through the implant cross-section (see Fig.5). Note the significant beam hardening artefact for Y-TZP equal for all four energies and representing as a significant reduction in gray value in the center of the implant image of approximately 50%.

**Figure 7:**
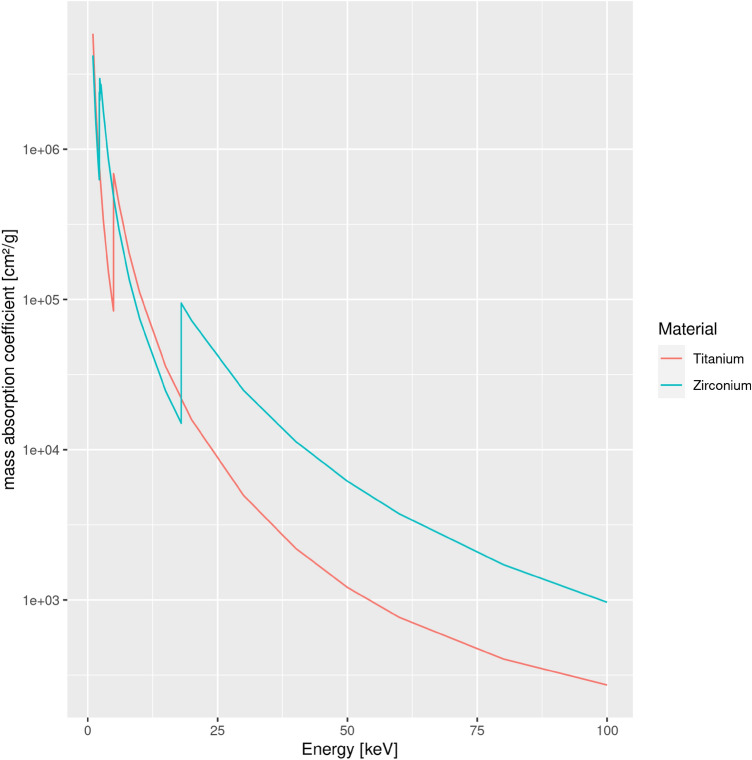
Plot of the mass attenuation coefficient for titanium and zirconium (as main compound of Y-TZP) for beam energies from 1 to 100 kVp (data taken from: physics.nist.gov).

The k-edge attenuation energy of Titanium is 4966.4 eV versus 17,997.6 eV for Zirconium as main compound of Y-TZP. It is probable that the spectrum emitted by the x-ray tube of the Accuitomo for the low energies (i.e. 60 kVp or 70 kVp) contains a large amount of x-rays in this energy range between 4 keV and 17 keV. From Fig. 7 it is evident, that from the k-egde of Zirconium for energies up to 100 keV the mass attenuation coefficient of the latter is considerably higher than that of Titanium with the curves running almost parallel to one another (Fig. 7). In this study, the profile-plot through the CBCT-reconstruction of the implant material shows the beam hardening effect by the centrally significantly lower gray values (Figs. 5 and 6). The latter is far more prominent for Y-TZP, where the gray value reduction centrally accounts for almost 50% of the peak value at the edge of the implant. Also, for Y-TZP there is only 4.9% difference between the central gray values when comparing 90–60 kVp. For titanium, this difference amounts to 39.8%.

## Discussion

The error induced by beam hardening is an incorrect representation of object composition (represented as gray values) along the projection (reconstruction) lines in the reconstructed volume. Clinically spoken, dark streaks occur along this direction which often compromise readability of the CBCT-images. The higher the atomic number, the more beam hardening will occur^15^. This work provides an analytical evaluation on the artefact-level caused by zirconia-implants (Y-TZP) versus pure titanium implants. Y-TZP composition as typical zirconium-oxide material with Yttrium-oxide additive was selected as representative for zirconia-implants. By using the 2D radiographic projection images acquired during a CBCT-scan as input for the assessment and the mathematical calculations, the attenuation and transmission caused by typical implant diameters (titanium: 4.0 mm, Y-TZP: 4.1 mm) was assessed and related to kilovoltage and material. For the typical peak kilovoltages (60–90 kVp) employed in dental CBCT-machines, up to 225% more attenuation of the x-ray-beam were observed for Y-TZP compared to titanium for which mass attenuation coefficients can be found in the National Institute of Standards and Technology website (physics.nist.gov) database. Unfortunately there is no such information easily available for zirconia. Hence, for a pure mathematical approach physical data are lacking and have to be derived from appropriate experiments. The approach of this experimental study was to use a subsample of the several hundred PROJs acquired in each CBCT-scan as input for the assessments. The rational behind this was that the manufacturers use these radiographs for the 3D reconstruction of the CBCT-volume. Hence it seems safe to assume that not overly much processing of these input images (which are normally not used or seen by the clinician) should be conducted. At least, it seems very likely that only linear operations are applied since the densities (representing energy incident on the detector) measured on the detector-pixels directly propagates into the inverse radon transform applied for 3D-reconstruction. Under this assumption the range between “no attenuation” as represented by only air images on the PROJs and the attenuation in the shadow of the implant was be defined as intensity range. Albeit physically not really correct, in the light of an unknown spectrum emitted by the source the simplified model used in this study allows for a reasonable modeling of the emitted intensity. Since the acquisition geometry of all PROJs was identical, the error induced by this simplification will be small. Plus, since the same error enters the equation (Eq. (2)) for all computations, the resulting error will be negligible for the comparative evaluation. The measured intensity “behind” an implant was computed as fraction of this intensity range. A relative attenuation coefficient $\mu$ was obtained from the linear attenuation coefficient (Eq. (2)) for each of the four exposure settings defined in Table 1. The difference in atomic numbers of the main implant-components (40 for the element zirconium versus 22 for titanium) obviously suggest that a zirconia-implant will absorb significantly more x-ray energy than titanium. However, Y-TZP is a rather complex mix of zirconium oxide (approximately 92%), yttrium oxide (approximately 5.5 %) plus some minor components (e.g. approximately 1.9% hafnium-oxide and 0.25% aluminum-oxide)^16^. Consequently, the entire mass attenuation coefficient of the compound certainly differs from that of the pure metal zirconium. In addition, the difference of the mass attenuation coefficient is not linearly related to beam-energy. Actual beam hardening in the reconstructed CBCT-images revealed approximately 50% difference between the artefacts occurring at 60 kVp versus 90 kVp for titanium. For the same energy range, however, for Y-TZP this maximum difference only amounts to approximately 5 %. This clearly supports the attenuation results, where Y-TZP attenuation only differed very slightly between 60 and 90 kVp (3.5% versus 4.5% intensity difference). These findings are also supported by the literature. Vasconcelos et al. for 70 kVp, 80 kVp and 90 kVp found significantly pronounced artefacts for zirconia when compared to a titanium implant^17^. For identical energies, the same relationship was reported by Freitas and colleagues^18^. From the experimental data it can be concluded, that beam hardening for Y-TZP in comparison to Titanium cannot be markedly reduced by higher voltages up to 90 kVp. This finding is also in agreement with the evaluation of the reconstructed CBCT-data (Fig. 5). For the clinician this outcome can be translated in such, that Y-TZP-artefacts cannot significantly be reduced by higher kilovoltages in the range applied in this evaluation. In how far kilovoltages above 90 kVp can reduce such artefacts cannot be directly concluded from this study. Yet the slope of the curve in Fig. 7 suggests that this effect at least holds true up to 100 kVp. For titanium, however, rather large attenuation differences of approximately 77% between 60 and 90 kVp were observed. In accordance with this finding beam hardening artefacts for titanium were significantly less pronounced in 90 kVp (Fig. 6). This experiment involves some shortcomings that require mentioning. First of all, unfortunately the Accuitomo device only employs an energy range between 60 and 90 kVpeak. Thus it was not possible to investigate higher energies up to 120 kVpeak which are also often used in dental CBCT machines^19^. On the other hand the approach required access to the raw projection radiographs which can be easily exported in this specific device while in most other CBCTs this is not the case. Considering the linear increase with kVpeak of transmission difference between Y-TZP and titanium, it seems safe to conclude that this will continue also in slightly higher kVpeak-energies. Also supporting this assumption is the fact that higher energies move the spectrum also further away from both k-edge attenuation energies. Another shortcoming of this study is the slight difference in diameter between the Y-TZP and the titanium implant. This was only due to availability of the respective implant-sample and the pure titanium rod representing a titanium implant. It should be noted, however, that this is only affecting the direct comparison of beam hardening artefacts in the reconstructed CBCT-images (Fig. 6) yet not the attenuation results which were calculated for exact the distances the “x-rays” traversing the implants.

## Conclusions

From the experiments and the mathematical modeling of the attenuation process we observed that Y-TZP-zirconia implants in the typical energy ranges employed in dental and maxillofacial CBCTs (60–90 kVp) will attenuate up to 225% more beam-energy when compared to pure titanium implants. For the energy spectrum used in this study, beam hardening caused by titanium can be reduced using higher energies while this is not the case for zirconia-ceramic (Y-TZP).

## Data Availability

The datasets used and/or analysed during the current study available from the corresponding author on reasonable request.
